# Autism spectrum disorder prevalence in Italy: a nationwide study promoted by the Ministry of Health

**DOI:** 10.1186/s13034-023-00673-0

**Published:** 2023-10-28

**Authors:** Maria Luisa Scattoni, Laura Maria Fatta, Martina Micai, Maria Enrica Sali, Marina Bellomo, Tommaso Salvitti, Francesca Fulceri, Angela Castellano, Massimo Molteni, Giovanna Gambino, Manuel Posada, Giovanna Romano, Maria Puopolo

**Affiliations:** 1 Research Coordination and Support Service, Istituto Superiore di Sanità, Rome, Italy; 2 IRCCS Eugenio Medea, Parini, Italy; 3 UOC ASP Palermo, Palermo, Italy; 4 ASL Roma 1, Rome, Italy; 5 Instituto de Salud Carlos III, Madrid, Spain; 6 Ministero della Salute, Rome, Italy; 7 Department of Neuroscience, Istituto Superiore di Sanità, Rome, Italy

**Keywords:** Autism spectrum disorder, Epidemiology, Prevalence estimate, Multistage screening

## Abstract

**Background:**

This nationwide study aimed to estimate Autism Spectrum Disorder (ASD) prevalence in 7–9-year-old Italian children. Promoted by Italy's Ministry of Health and coordinated by the National Observatory for Autism at the National Institute of Health, it covered schools in northern (Lecco and Monza-Brianza), central (Rome and its province), and southern (Palermo and its province) regions from February 24, 2016, to February 23, 2018, using a multi-stage approach defined by the European Union's ASD network.

**Methods:**

Phase one identified ASD-diagnosed children in mainstream schools through local Ministry of Education (MoE) disability registries. Phase two had a subset of schools screen 7–9-year-olds using the Social Communication Questionnaire-Life version (SCQ-L). Those with SCQ-L scores of 15 + underwent clinical consultation for ASD symptoms, cognitive abilities, and life skills. To counter potential false negatives, 20% scoring 11–14 were randomly assessed via Autism Diagnostic Interview-Revised (ADI-R).

**Results:**

MoE data revealed 9.8 per 1000 certified ASD children in the north, 12.2 in the central, and 10.3 in the south. In phase two, 35,823 SCQ-L questionnaires were distributed across 198 schools (northern: 11,190 in 49 schools, central: 13,628 in 87 schools, southern: 11,005 in 62 schools). Of SCQ-L respondents, 2.4% (n = 390) scored above the 15 cutoff. Among these, 100 had ASD diagnoses, and 50 had other diagnoses. Among 115 families assessed, 16.5% (n = 19) received ASD diagnoses.

**Conclusions:**

The estimated prevalence of ASD in Italy was 13.4 (11.3–16.0) per 1,000 children aged 7–9 years, with a male-to-female ratio of 4.4:1. It will guide national policies in enhancing services tailored to the specific needs of autistic children.

## Key Practitioner Message

1. Our study reveals ASD prevalence in Italian 7 to 9-year-olds, guiding resource allocation and interventions across regions.
2. Using a trusted multi-stage approach, our findings offer practitioners accurate data for informed decision-making and policy formulation.
3. SCQ-L screening guarantees early identification of autistic children (Level 1).
4. Our study's ASD prevalence estimates of 1 per 77 children equips practitioners to advocate for tailored policies, ensuring specialized support for autistic children and families.

## Introduction

Autism Spectrum Disorder (ASD) is a neurodevelopmental condition characterized by impairments in socio-relational domains and both verbal and nonverbal communication skills, as well as the presence of restricted, repetitive, and stereotypical interests and activities [1]. ASD is a diverse condition encompassing individuals with varying support needs and associated psychiatric and medical co-occurring conditions.

In recent years, there has been an observed increase in the prevalence of ASD. This rise can be attributed to several factors, including improved availability of diagnostic services, and increased public awareness among laypeople and professionals [14]. Additionally, the expansion of diagnostic criteria and assessment tools for ASD has contributed to the identification of individuals with the condition [15].

Epidemiological studies on ASD have significantly increased in recent years; however, they often yield varying and conflicting results. This can be attributed to numerous complex factors, including differences in the abilities of clinicians to identify the condition, variations in the screening tools utilized, diverse models of conceptualization and categorization of ASD, discrepancies in methodological protocols, and variations in sample recruitment [36]. Furthermore, the introduction of the Diagnostic and Statistical Manual of Mental Disorders, Fifth Edition [1], may have adjusted the threshold for clinical diagnosis, potentially leading to an increased prevalence of individuals diagnosed with a milder phenotype of ASD who would have previously remained undiagnosed [26]. Lastly, the contribution of environmental risk factors cannot be excluded when considering the increase in ASD prevalence [21].

According to a comprehensive review of studies worldwide, the estimated overall prevalence of ASD ranges from 1.1 per 10,000 to 436.0 per 10,000, with a calculated average of 100 per 10,000 [43]. In the United States, the Autism and Developmental Disabilities Monitoring (ADDM) Network reported an overall prevalence estimate of 27.6 per 1000 children aged 8 years in 11 states in 2020, equating to a ratio of 1 in 36 children [24]. In Europe, several studies have been published, including four in Italy [9]. The first one was part of the Autism Spectrum Disorder in the European Union (ASDEU) project and estimated a prevalence in the city of Pisa of 11.5 per 1000 children aged 7 to 9 years, using probabilistic calculations to adjust for non-responses [28]. Lower prevalence rates for children aged 6–10 years were reported by the Child and Adolescent Mental Health Units of two regional healthcare information systems: SMAIL in Piemonte (4.2 per 1000) and ELEA in Emilia-Romagna (4.3 per 1000) regions (as reported in the National Guidelines approved by the National-Regions Conference by Atti n. 53/CU of 10/05/2018) [9, 10]. Lastly, a prevalence study conducted in the Abruzzo region, baministrative data of the Autism Regional Reference Center, estimated a prevalence of 8.0 per 1000 in children aged 6 to 8 years [38]. Due to the lack of an Italian ASD prevalence estimate and of a national health information system or ASD registry, the Italian Ministry of Health funded this comprehensive population-based study.

The present study aimed to estimate the prevalence rate of ASD in Italian children aged 7–9 years. The study was conducted by the National Observatory for ASD, coordinated by the National Institute of Health (Istituto Superiore di Sanità, ISS), using a modified version of the ASDEU project protocol. This protocol was the result of the ‘European Protocol for Autism Spectrum Disorder Prevalence [31] within the European Autism Information System (EAIS) project aimed to facilitate a common format to be tested in the European Member States to determine the ASD prevalence in the EU. The prevalence of ASD was measured in three geographical areas: North, Central, and South, encompassing both urban and rural residence of the school. This study is the first to provide an Italian ASD prevalence estimate based on a large sample size conducted across different geographical areas throughout the country. In addition, for the first time, this study collected data records from the Italian Ministry of Education (MoE) and screened all children within the target age group attending mainstream schools using the Social Communication Questionnaire—Lifetime version (SCQ-L) [33] completed by their parents. A similar ASDEU study conducted in Denmark, Finland, Iceland, and France collected ASD prevalence data using nationwide health registry systems and population-based regional registries [12]. In our study, although on a smaller scale, we identified children diagnosed with ASD in the three geographical areas by accessing the data records from the MoE. To ensure comprehensive case identification, we combined access to the MoE records with population screening, which is less influenced by bias from clinical, educational, or registered records, and is considered a high research standard. The goal was to support the Italian government with reliable data for defining suitable health, social, and educational services and public health policies tailored and targeted to the specific needs of individuals with ASD and their families.

## Methods

### Prevalence protocol study design

The present ASD prevalence study, conducted between February 24th, 2016, and February 23rd, 2018, was promoted by the Italian Ministry of Health in collaboration with the MoE and coordinated by the National Observatory for ASD. The study utilized an adapted version of the ASDEU project protocol. The protocol for the study was approved by the Ethic Committee of the ISS on March 15th, 2016.

### Geographical areas selection and rural/urban schools’ invitation

Three geographical areas were selected according to the following criteria established by the ASDEU project: a. well-defined and delimited geographical and administrative area (s); b. stable population; c. compulsory education system at the ages of the study subjects; d. existence of a Public Health Care System covering nearly 100% of the population; e. accessibility of data from educational and special educational sources; f. no potential selection bias due to the existence of reference services of ASD diagnosis, treatment, or special education facilities, which are located outside the area but close enough for children living within the area to access—this could result in missing children within the study area.; g. accessibility to cases’ clinical records; h. data accessibility from clinics and institutional private services; i. rural and urban residence of schools should be considered. Finally, ASD representative organizations and other regional stakeholders must be involved in the study. By adhering to these criteria, the study aimed to ensure a comprehensive and representative assessment of ASD prevalence within the chosen geographical areas.

The selected three geographical areas were: the city of Lecco and the area of Monza-Brianza (North area), the city of Rome and its province (Center area), and the city of Palermo and its province (South area). Each of these areas had a national clinical referral center specializing in the diagnosis of ASD in children (Lecco-Monza-Brianza: IRCCS E. Medea, La Nostra Famiglia, Bosisio Parini; Rome and its province: ASL Roma 1- 'La Scarpetta'; Palermo and its province: ASP Palermo). In adherence to the ASDEU protocol, both rural and urban schools were invited within each of the three areas. This ensured a representation of different educational settings and demographics within the study population.

### Population

#### Sample size calculation

The sample size was determined by considering two key factors: (1) estimated target population: children between the ages of 7 and 9 years who were residents of the areas during the study period (children born between January 1st, 2007, and December 31st, 2009); (2) expected number of ASD cases based on existing data or previous studies on ASD prevalence rates. By combining information on the estimated target population and the expected number of ASD cases, the researchers were able to determine an appropriate sample size that would provide statistically reliable results for estimating the prevalence rate of ASD in the areas and age group.

According to the National Institute of Statistics (ISTAT) [20] data, in the northern area, the estimated target population of 7–9-year-olds as of January 1st, 2017, was approximately 26,066 children in Monza Brianza and 9971 children in Lecco. This totals to an estimated population of 36,037 children in the northern area. In the center area, which includes Rome and its province, the estimated target population of 7–9-year-olds was 124,346 children. In the southern area, which includes Palermo and its province, the estimated target population of 7–9-year-olds was 37,632 children.

At the time of the study, the expected number of ASD cases was approximately 1% based on previous research [13]. The precision of this estimation was around 0.2 with a 95% confidence level. To obtain reasonably accurate estimations, the ASDEU protocol recommended selecting a sample size of 8,000–10,000 children within the specified age range. Considering an estimated dropout rate of 20%, a sample size of 12,500 children per area was calculated. This size would help ensure that enough data were collected to accurately estimate the prevalence of ASD in the three areas.

### Procedure

The present study was divided into two phases: phase (1) identification of children already certified with ASD from the MoE. These records provided information on children who had previously received a formal diagnosis of ASD and were already recognized by the educational system as having the condition; phase (2) screening and identification, by clinical assessment, of ASD cases not registered with the MoE. Parents of children in the target age range (7–9 years old) attending mainstream schools have been invited to fill in the SCQ-L. Children who scored equal or above the cut-off point of 15 (≥ 15) on the SCQ-L were invited to undergo an expert clinical consultation for a comprehensive assessment of ASD symptoms, as well as cognitive and daily life skills. This phase aimed to identify children with ASD who may not have been previously diagnosed or certified.

### Phase 1: Certified ASD diagnoses obtained from the Ministry of Education's records

In Italy, students with ASD or other disabilities requiring special educational support are assisted in their learning and daily living skills, as well as in developing relationships with their typically developing peers, by support teachers and/or educators, in accordance with Italian Law 104/1992 (Gazzetta Ufficiale della Repubblica Italiana) [18]. The MoE maintains the unique regional register of children with disabilities, including those with ASD per se or with other comorbidities, requiring health and educational support. To be included in the registry, parents of ASD children present at the MoE the clinical certification of diagnosis produced by a child psychiatry unit of the Nation health system following a multidisciplinary evaluation with standardized tools. Unfortunately, diagnoses are included in the MoE registry either with the ICD-9, ICD-10, DSM-IV-TR and DSM 5 diagnostic codes.

In the present study, access to the number of ASD diagnoses in the MoE's Office for Disability registry was made possible through an agreement with the ISS. A designated contact person was assigned by the MoE’s regional/local offices for Disability in each of the three areas. This contact person assisted the ISS researchers in establishing initial contact with the school deans. The MoE’s regional/local officers for Disability provided to ISS records of children born between January 1st, 2007 and December 31st, 2009 (aged 7–9 years), who were attending primary mainstream schools in the three areas and had received a diagnosis and certification of disability.

Subsequently, the ISS's clinical team reviewed all provided records to identify children diagnosed with ASD: (1) Diagnostic criteria from the DSM-5 [1]; (2) International Classification of Diseases [41] codes 299.0, 299.80, and ICD-9 nosographic labels F84.0, F84.5, F84.8, F84.9; (3) DSM-IV-TR [2]. In cases where conflicting information was found in the records, the local Child Psychiatry Unit responsible for the diagnosis and disability certification was contacted for clarification and resolution.

### Phase 2: Screening of the general population

The second phase of the present study involved the screening for ASD among the general population of 7–9-year-old children born between January 1st, 2007, and December 31st, 2009, in invited schools. Children who scored equal or above the cut-off 15 on the SCQ-L were further assessed for ASD diagnosis.

The ISS and the MoE compiled a list of schools in the three selected areas. As per the ASDEU project requirements, local and national stakeholders were contacted to promote the study. The MoE regional/local officers sent official letters to the deans of the schools, inviting their participation, and explaining the study's aims, as well as its public health and scientific significance.

During school visits, the study protocol was presented by the ISS researchers and MoE regional/local officers in the presence of the school's dean, teachers, and parents. Parents of the 7- to 9-year-old children attending the invited schools received an envelope containing a letter explaining the purpose of the study, an informed consent form, and the SCQ-L.

The SCQ-L, a validated tool for ASD screening, consists of 40 yes/no response items completed by parents or primary carer. It assesses language abilities, presence of siblings with ASD, social-relational development at 4–5 years, and atypical behaviors at the time of assessment. A cut-off score of 15 was selected, as it is commonly considered suggestive of ASD presence [8, 33].

Two weeks after the delivery of the envelopes, the ISS team collected the sealed envelopes containing the completed SCQ-L questionnaires and signed informed consent forms. The signed informed consent was required for the analysis of the SCQ-L data.

#### Clinical assessment procedure

In cases where the child’s SCQ-L score was equal to or greater than 15, the ISS team invited the child and their parent to the selected reference center for ASD. The clinical team consisted of a child psychiatrist from each area's center and a psychologist from the ISS. The assessment of the children was conducted at the clinical center using the Autism Diagnostic Interview-Revised (ADI-R) [34, 35], Autism Diagnostic Observation Schedule-2 (ADOS-2) [23], and clinical judgment, following the criteria outlined in the DSM-IV-TR and DSM-5.

The ADI-R is a comprehensive 93-item interview that covers the individual's developmental history and focuses on three functional domains: language and communication, reciprocal social interactions, and restricted, repetitive, and stereotyped behaviors and interests. ADOS-2 is a semi-structured and standardized assessment with four modules based on the individual's level of expressive language ability. It includes play-based activities to gather information on communication, reciprocal social interactions, and restricted and repetitive behaviors associated with an ASD diagnosis. The ADOS-2 allows for comparison across modules through an algorithmic scoring system, ensuring consistent scores regardless of the module used. For this study, module three, designed for children with fluent language skills, was administered, while module two was used for children with limited language abilities. Algorithmic scores were calculated for all participants.

The intellectual abilities and cognitive functions of verbal children were assessed using the Wechsler Intelligence Scale for Children-IV (WISC-IV) [30, 39], which evaluates verbal comprehension, perceptual reasoning, working memory, and processing speed. Nonverbal children's cognitive abilities were assessed using the Leiter international performance scale-revised (Leiter-R) [33], which targets nonverbal intelligence in fluid reasoning, visualization, visuospatial memory, and attention. The brief form of the Leiter-R, including subdomains such as Figure Ground, Form Completion, Sequential Order, and Repetitive Pattern, was administered to obtain a standardized nonverbal Brief Intelligence Quotient (IQ). Each subtest and brief IQ score demonstrated excellent validity and reliability [32].

Furthermore, parents were interviewed using the Vineland Adaptive Behavioral Scales (VABS) [5, 37], a standardized parent interview consisting of 297 items that assess daily skills in four domains of functioning: Communication Skills, Daily Living Skills, Social Skills, and Motor Skills. Equivalent ages based on published Italian norms were derived from the adaptive behavior composite score for each domain.

Upon receiving an ASD diagnosis or any other diagnosis, the clinical team developed a specific and personalized recommendations/interventions plan for the child and their family.

To minimize the risk of false negatives, the ISS team randomly selected the 20% of children who scored between 11 and 14 on the SCQ-L and invited their primary carer to participate in a semi-structured interview over the phone using the ADI-R, following the approach described by Carpenter and colleagues [7]. It has been suggested in previous studies to lower the conventional SCQ-L cut-off point of 15 when screening the general school population [27]. Some studies propose using a lower cut-off point of 11 [3, 17, 29, 40] or 12 [11] to improve sensitivity and reduce the likelihood of false negatives.

### Statistical analysis

The prevalence of ASD (reported as number of ASD cases per 1000 children) and its 95% confidence interval (CI 95%) were estimated in the present study, both overall and stratified by geographical areas (North, Center, South), SCQ-L score classes (≥ 15, 11–14, < 11), gender (male *vs* female), and residence of the school (rural *vs* urban). Participants who did not complete the SCQ-L or did not provide a signed informed consent were excluded from the estimation.

ASD cases were counted independently from the source of data for diagnosis (MoE records –phase 1– or clinical screening –phase 2). The survey data commands (svyset) in Stata 15 were used to specify the survey design by setting weights for participants and SCQ-L classes as strata. For children with a certified diagnosis in the MoE database (phase 1), a weight of 1 was assigned. For children who underwent clinical screening (phase 2), weights were calculated based on the reciprocal of the probability of being clinically screened. For children with SCQ-L scores in the range of 11 to 14, weights accounted for 20% sample. These weights were calculated separately for each geographical area and SCQ-L score class, by accounting for the certified ASD diagnoses in the MoE database and the number of children who underwent clinical screening.

Prevalence estimates were obtained using the "proportion" command, considering the specified survey settings. The statistical analyses were conducted using Data Analysis and Statistical Software STATA (version 15.1; Stata Corp., College Station, TX, USA).

## Results

### Phase 1: Certified ASD diagnosis records of the Ministry of Education

The results of the study showed a percentage of children certified with ASD retrieved from the MoE records of 10.9 per 1,000 children (95% CI 9.8–12.1). For each geographical area, the North had a percentage of 9.8 per 1,000 children (n = 110, 95% CI 8.1–11.8) children certified with ASD, the Center 12.2 per 1,000 children % (n = 167, 95% CI 10.5–14.2), and the South 10.3 per 1,000 children % (n = 113, 95% CI 8.5–12.3). Table 1 summarizes process and results.

**Table 1:** Summary of the steps and results of phase 1 and 2

		North *(n) (%)*	Center *(n) (%)*	South *(n) (%)*
**Phase 1**				
MoE partecipation	ASD diagnosis records	110	167	113
**Phase 2**				
Schools partecipation	Invited schools	49	87	62
	Participating schools	32 (65.3)	42 (48.3)	50 (80.6)
Parents partecipation	Invited parents	**11,190**	**13,628**	**11,005**
	SCQ not filled	4308 (38.5)	8631 (63.3)	6584 (59.8)
	Filled SCQ, not valid score	6 (0.05)	–	1(0.009)
	Filled SCQ, valid score	6876 (61.4)	4997 (36.7)	4420 (40.2)
Scoring SCQ and clinical evaluation	SCQ ≥ 15	**153 (2.2)**	**117 (2.3)**	**120 (2.7)**
	ASD already certified	29 (18.9)	30 (25.6)	41 (34.2)
	Diagnostic assessment	18 (11.8)	37 (31.6)	60 (50.0)
	ASD not registered with MoE	7 (4.6)	8 (6.8)	4 (3.3)
	New NDD diagnosis	3 (2.0)	19 (16.2)	19 (15.8)
	11 ≤ SCQ ≤ 14	**358 (5.2)**	**216 (4.3)**	**239 (5.4)**
	ASD already certified	11 (3.1)	2 (0.9)	4 (1.7)
	20% assessed	64 (17.9)	35 (16.2)	36 (15.1)
	ASD not registered with MoE	0 (0.0)	0 (0.0)	0 (0.0)

Percentages are calculated as number of schools/parents/subjects (numerator) on total number (in bold) of invited schools/parents/children

### Phase 2: Screening of the general population

#### School’s rate participation

**Fig. 1:**
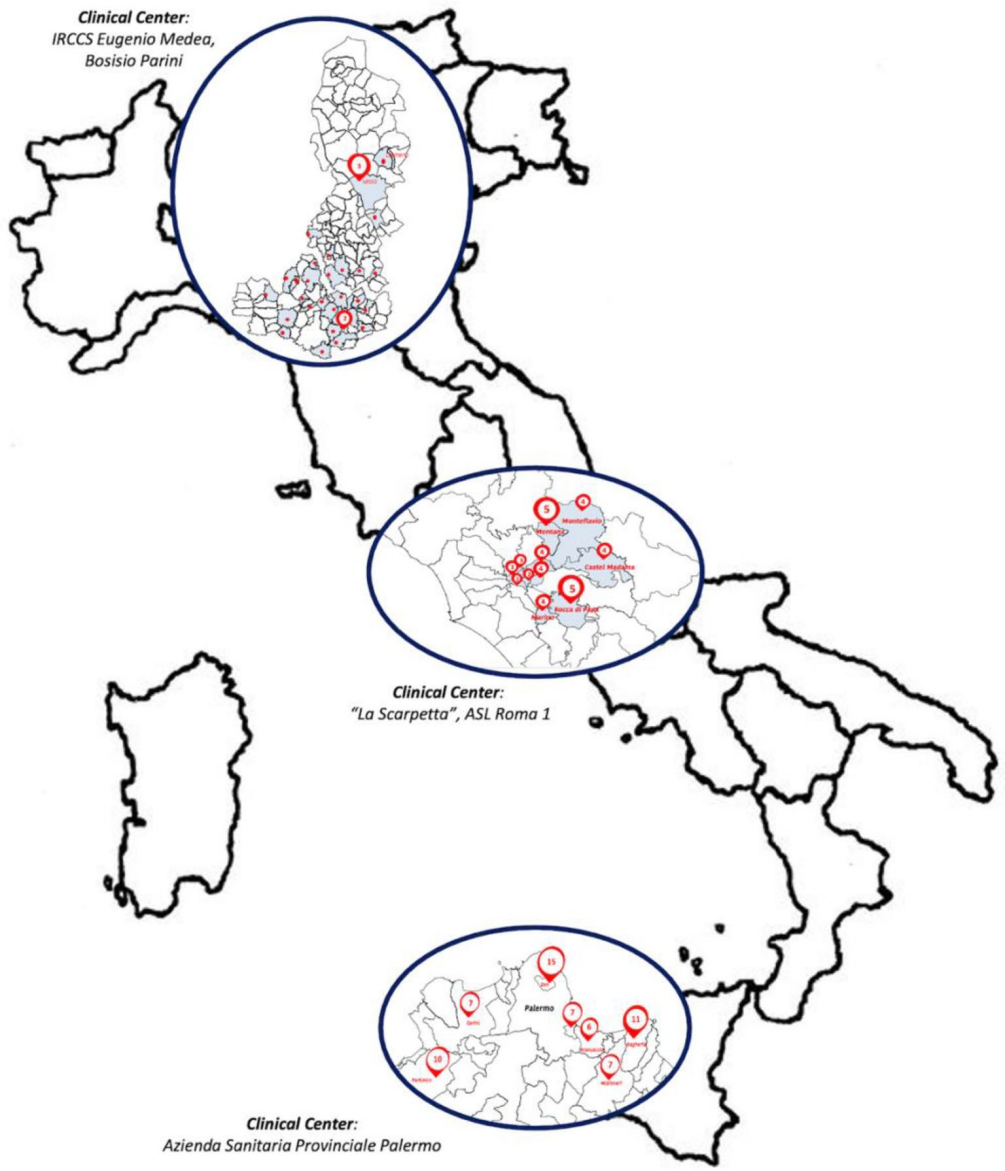
Map of the areas selected including the location of schools and clinical referral centers

To achieve the desired sample size of 12,500 children in each area, ISS and MoE randomly selected and invited 27.8% (n = 198) of schools, equally distributed in the rural and urban areas, out of 711 schools present in the three areas (Fig. 1). Specifically, 49 schools were invited (community sample of 25,414 children), out of 96 in the North Area. In the Center area, 87 schools were invited (community sample of 14,116 children) out of the 499 schools present. In the South area, 62 schools were chosen out of the 116 schools (community sample of 27,841) present in the territory.

Of the 198 schools invited to participate in the project, 62.6% (n = 124) agreed to take part. In the North area, the school participation rate was 65.3%, indicating that 32 out of the invited 49 schools agreed to join the project. In the Center area, the participation rate was 48.3%, with 42 out of the invited 87 schools agreeing to participate. The southern area achieved the highest participation rate, with 80.6% (n = 50) of the schools (out of the total 62 schools) joining the project.

#### Parent’s rate participation

Out of 35,823 parents who received the SCQ-L, 45.5% (16,293 parents) agreed to participate by completing the SCQ-L and signing the consent form. The highest participation rate was observed in the Northern area: out of 11,190 parents who received the SCQ-L, 61.4% (6876 parents) agreed to participate. In the Central area, out of 13,628 parents who received the SCQ-L, 36.7% (4997 parents) agreed to participate by filling out the SCQ-L and signing the consent form. In the South area, out of 11,005 parents who received the SCQ-L, 40.2% (4420 parents) joined the project.

#### SCQ-L screening results

The SCQ-L scores were calculated for a population of 16,293 children, resulting in a mean score of 4.58 (SD = 3.95; range: 0–36). Out of the 390 children with an SCQ-L ≥ 15 score, 100 were already certified with ASD before the present study. The remaining 289 children were candidates to be assessed by clinicians.

More than a half of the parents (174 out of 289, 60.2%) refused the child’s assessment. The distribution of refusals was not equal among the different areas, with the North area having the highest refusal rate (n = 106, 60.9%), followed by the Center area (n = 50, 28.7%), and the South area (n = 18, 10.3%).

Among the remaining 115 children (North: 18, Center: 37, South: 60) assessed, 50 of them had already been diagnosed with other conditions such as Down syndrome, Williams syndrome, and other neurodevelopmental disorders (NDD). Despite their existing diagnoses, these children were still included in the assessment process. Out of the 115 children assessed, a total of 19 diagnoses of ASD not registered with the MoE and 41 diagnoses of other NDD were made. The distribution of these diagnoses was as follows: North (ASD: 7; NDD: 3), Center (ASD: 8; NDD: 19), and South (ASD: 4; NDD: 19). The remaining 55 children (North: 8; Center: 10; South: 37) were identified as typically developing. 20% of the children with a SCQ-L score between 11 and 14 were randomly selected and assessed to minimize false negatives, resulting in 135 children (64 in the North, 35 in the Center, and 36 in the South). No diagnosis of ASD or other NDDs was made in these children. ISS and MoE selected schools randomly, ensuring an equal distribution between rural and urban areas, in accordance with the ASDEU protocol. We do not possess data regarding the geographical locations of schools that have or have not complied. However, we conducted an analysis to examine the geographical distribution of children in urban and rural areas. In the SCQ classes > 15 and 11–14, there was no significant difference in the percentage of certified children, the percentage of children assessed among those to be assessed, or the percentage of new ASD diagnoses between urban and rural children. Additionally, in the SCQ class < 11, the percentage of certified children did not vary significantly between urban and rural children.

#### Clinical assessment results

The 19 children (6 females) diagnosed with ASD showed a mean SCQ-L score of 19.26 (SD = 3.21, range = 15–25). Findings pertaining to the clinical assessment of core symptoms revealed a mean ADOS-2 calibrated severity score of 5.0 (SD = 1.4, range = 2–8), a mean ADI-R Communication score of 8.9 (SD = 3.3 range 2–15), a mean ADI-R Reciprocal Social Interaction score of 12.4 (SD = 3.4, range 8–18), a mean ADI-R Restricted Stereotyped Behaviors score of 4.89 (SD = 2.0 range 2–10), a mean ADI-R Abnormality of Development Evident at or Before 36 Months score of 1.89 (SD = 1.1 range 0–4).

Cognitive functions were also assessed, resulting in a mean WISC-IV Total score of 95.29 for 17 children (SD = 25.7, range = 46–132). The cognitive assessment of two children with intellectual disability was conducted using the Leiter-R (mean = 71.5; SD = 9.19, range = 65–78). Furthermore, the mean of VABS Age Equivalent scores (AEs) adaptive functioning available for 18 children was in Communication score of 7.7 (SD = 2.1 range 4.6–12), daily living skills score 6.5 (SD 2.5 range 3.4–10.7), Socialization score 5.2 (SD = 1.9 range 1.1–9.1).

The majority of children diagnosed with ASD (and not registered with the MoE) were, at the time of the evaluation, between 8 and 9 years old (mean age = 8.7, SD = 0.9, range: 7.3–10.3). Among those children, 57.9% (n = 11) required substantial support (level 2, DSM-5), while 42.1% (n = 8) required support (level 1, DSM-5). Co-occurring conditions were also identified. Individual scores are presented in Table 2.

**Table 2:** Clinical assessment results of the children diagnosed with ASD and not registered with the MoE

Subject				Diagnostic assessment												Final diagnosis
				SCQ-L	ADOS				ADI-R			IQ	VABS AEs			
*N°*	*Age/Gender*	*Area/Setting*	*Incoming diagnosis*	*Score*	*Social Affect*	*RRB*	*Total*	*Severity*	*Social Interaction*	*Communication*	*RRB*	*Score*	*Communicative*	*Daily*	*Social*	
1	8.2/M	North/R	–	22	5	3	8	5	9	12	5	121	8.1	6.1	5.5	ASD level 2
2	9.5/M	North/U	ID + LaD	21	12	2	14	8	15	12	6	49	5.8	3.5	1.1	ASD level 2 + ID
3	10.0/M	North/U	ADHD + LD	20	5	2	7	4	15	8	3	112	8.8	8.1	3.9	ASD level 1 + ADHD + LD
4	9.3/M	North/U	LaD + OCD	20	1	3	4	2	10	7	8	104	9.10	9.9	6.1	ASD level 1 + LD + OCD
5	7.5/F	North /U	–	18	8	0	8	5	10	7	2	102	8.1	5.1	5.1	ASD level 2
6	8.0/M	North/U	–	16	6	2	8	5	9	9	3	81	6.6	4.8	4.1	ASD level 2
7	8.2/F	North/U	LaD	22	6	1	7	4	13	13	5	84	4.6	4.0	3.4	ASD level 1 + LD
8	10.1/F	Center/R	ADHD	25	6	2	8	5	18	10	7	103	5.4	4.1	3.5	ASD level 2
9	9.1/M	Center/R	DCD	24	6	3	9	6	18	7	6	93	6.9	3.4	3.8	ASD level 2
10	9.9/F	Center/R	ID + DCD	24	8	2	10	6	18	9	3	46	6.8	6.3	4.1	ASD level 2 + Mild ID
11	10.3/M	Center/R	CD	21	9	2	11	7	16	15	10	117	10.7	10.7	5.3	ASD level 2
12	9.0/M	Center/U	CD + DCD	19	8	2	10	6	8	13	3	77	9.7	10.7	6.5	ASD level 2
13	8.4/M	Center/U	–	19	8	1	9	6	10	5	6	132	10.3	9.10	5.9	ASD level 2
14	7.8/M	Center/U	–	17	5	2	7	4	9	2	5	65	–	–	–	ASD level 1
15	7.6/M	Center/U	–	16	7	1	8	5	9	5	6	90	12.0	9.4	5.9	ASD level 1
16	8.5/M	South/R	TS	16	8	2	10	6	12	9	5	65	5.5	4.7	5.1	ASD level 2
17	8.11/M	South/U	–	15	5	2	7	4	13	7	3	122	6.6	5.5	8.2	ASD level 1
18	7.3/F	South/U	–	15	5	1	6	4	13	12	3	78	6.10	6.0	6.8	ASD level 1
19	9.0/F	South/U	–	16	6	0	6	3	11	8	4	122	7.2	5.9	9.1	ASD level 1

*ADHD* Attention Deficit Hyperactivity Disorder,* ADI-R* Autism Diagnostic Interview-Revised,* ADOS-2* Autism Diagnostic Observational Schedule, 2nd Edition,* ASD* Autism Spectrum Disorder,,* CD* Conduct Disorder,* DCD* Developmental Coordination Disorder,* ID* Intellectual Disability,* IQ* Intellectual Quotient in total score,* LD* Learning Disability,* M* Male,* F* Female,* LaD* Language Disorder, *OCD* Obsessive-Compulsive Disorder, *RRB* Repetitive and Repetitive Behaviors,* R* Rural setting,* U* Urban setting,* SCQ* Social Communication Questionnaire-Lifetime Version, *TS* Tuberous Sclerosis*, VABS AEs* Vineland Adaptive Behavioral Scale, age equivalent scores

All subjects were assessed using the Wechsler Intelligence Scale for Children, IV edition with exception of subjects n°16 and n°18 who performed the Leiter international performance scale-revised

#### ASD prevalence

Based on the information provided by phase 1 and 2, prevalence of ASD was 13.4 (11.3–16.0).

When stratifying by gender, a higher prevalence of ASD was observed in males (22.2 per 1,000 children, 95% CI 18.3–26.8) compared to females (5.1, 95% CI 3.0–9.0), with a male-to-female ratio of 4.4:1. Prevalence estimates were consistent across the three geographical areas, as well as across rural (12.4 per 1000 children, 95% CI 9.68–15.96) and urban (14.2, 95% CI 10.8–18.5) residence of school. Furthermore, as expected, there was a higher prevalence of ASD in children with SCQ-L scores ≥ 15 (443.2 per 1000 children, 95% CI 358.3–531.56) compared to those with SCQ-L scores ranging from 11 to 14 (20.9, 95% CI 12.7–34.2) and SCQ-L scores below 11 (1.9, 95% CI 1.3 -2.8).

Estimates were consistent among geographical areas. The prevalence of the North area was 15.4 per 1000 (95% IC 11.4–20.8), 1:66 or 15:1,000, with a male to female gender ratio of 3.1:1 (male: 22.7, 95% IC 16.0–32.1; female: 7.4, 95% IC 3.3–16.5). No difference was observed for residence of school (urban: 17.1 per 1000 children, 95% IC 11.9–24.6; rural: 11.0, 95% IC 5.4–22.1). Higher prevalence of ASD was observed in children with a SCQ-L ≥ 15 group (504.7 per 1,000 children, 95% IC 320.3–687.8) compared to those with 11 ≥ SCQ-L ≤ 14 (30.7, 16.4–56.9) and SCQ-L < 11 (2.8, 95% IC 1.7–4.5). The ASD prevalence of children living in the Central area resulted in 12.2 per 1,000 children (95% IC 9.7–15.3), 1:83 or 12:1,000, with a male to female ratio of 5.3:1 (male: 20.5, 95% IC 15.9–26.5; female: 3.9, 95% IC 1.7–8.6). No difference was observed for residence of school (urban: 10.3, 6.7–15.7; rural: 14.1, 10.0–19.8). Higher ASD prevalence for children with a SCQ-L ≥ 15 score (417.2, 95% IC 301.4–542.8), compared to those with 11 ≥ SCQ-L ≤ 14 (9.3, 95% IC 2.2–38.1) and SCQ-L < 11 (2.1, 95% IC 1.2–4.0) was observed. The prevalence of children with ASD in the Southern area was 11.8 per 1,000 children (95% IC 9.5–14.6), 1:83 or 12:1,000, with a male to female ratio of 7.8:1 (male: 23.4, 95% IC 18.5–29.6; female: 3.0, 95% IC 1.3–7.2). No difference was observed for settings (urban: 11.8, 95% IC 8.2–17.0; rural: 11.8, 95% IC 8.5–16.5). Higher ASD prevalence for children with a SCQ-L ≥ 15 score (390.6, 95% IC 302.1–487.1), compared to those with 11 ≥ SCQ-L ≤ 14 (16.7, 95% IC 5.9–46.2) and SCQ-L < 11 (0.2, 95% IC 0.0–1.7) was observed.

## Discussion

The present study, implemented as part of a national strategy of the Italian Ministry of Health, was carried out by the National Observatory for Autism (ISS) in collaboration with the MoE with the aim of determining the prevalence of ASD in Italy. The findings revealed a prevalence rate of 1 in 77 children aged 7–9 years old between 2016 and 2018. Our study protocol exhibited several notable strengths when compared to previous research conducted in Italy. It encompassed three distinct and sizable geographic areas in the North, Center, and South of Italy, including both rural and urban areas. Furthermore, the study benefited from a substantial sample size, with a total of 16,293 SCQ-L respondents out of the 35,823 individuals invited, corresponding to a response rate of 45.0%.

The present study followed the ASDEU methodological criteria and was conducted in two phases. In the first phase, we accessed the MoE registry to identify children with certified ASD who were already enrolled in mainstream schools within the selected areas. The second phase involved screening all children in the invited schools using the SCQ-L and assessing those who obtained a score equal to or higher than 15 on the SCQ-L for ASD symptomatology, cognitive abilities, and daily skills. This study protocol allowed us to capture data on both children who had already received an ASD diagnosis and had access to educational (e.g., support teacher, educator, individualized learning program, personalized test, and periodical learning reports by teachers) and healthcare services (Phase 1) and children who had not yet been diagnosed (Phase 2).

The present project presented several advantages compared to previous ASDEU studies conducted in Italy [28] and Spain [16]. Firstly, our study was carried out in three extensive geographical areas, including schools from both urban and rural residence. The study was planned by enrolling schools distributed both in rural and urban areas. Overall, analyses found no significant differences in the percentages of certified children, the percentages of children assessed among those who were to be assessed, or the percentages of new diagnoses in assessed children among rural and urban areas, across all SCQ classes. These findings strengthen our estimate, as bias due to geographical localization (urban vs rural) may likely be excluded. This differed from previous studies that focused only on the metropolitan area of Pisa (Italy) and the County of Gipuzkoa (Spain). Secondly, our study considered the unique challenges faced by children in rural areas, as evidence suggests that they are at a higher risk of receiving a late or missed diagnosis. Moreover, accessibility to services has a significant impact on epidemiological rates, and our study accounted for this by including both urban and rural areas, as greater service availability facilitates early screening and diagnosis of ASD. Lastly, in the school population screening stage, our study involved testing all children attending the invited schools using the SCQ-L, whereas Narzisi and colleagues [28] identified children for further evaluation based on the Teacher Nomination form (TN) filled out by teachers and then screened them using the SCQ-L.

Our study’s prevalence rate of 13.4/1000 was higher compared to other Italian studies conducted in the Piemonte region (4.2/1000), Emilia-Romagna region (4.3/1000), and Abruzzo region (7.8/1000) according to the administrative regional reports (reported in the Ministry of Health Guidelines approved by the National-Regions Conference, May 10th 2018) [10] and the study by Valenti and colleagues [39]. These differences in the estimate of prevalence rates could be attributed to the different methods employed. In addition, our estimate may be an overestimate given the low rate of parental participation; parents with children at higher risk or with greater signals picked up by parents may have been more likely to have responded. Generally, prevalence rates obtained from registries tend to be lower compared to rates measured through screening the general population. Registry-based prevalence may miss individuals who do not have access to services or those who are undiagnosed.

In addition, our study reported a higher ASD prevalence compared to the ASDEU study conducted in Pisa (11.5/1000; [28]). This difference could potentially be attributed to the method used for selecting children to be assessed with the SCQ-L. In the Pisa study, children were selected based on the TN completed by teachers, which could introduce a selection bias. This selective approach may lead to an underestimation of ASD prevalence as it could miss children who may not exhibit obvious signs or have not been identified by teachers. In contrast, our study employed a broader screening approach, testing all children attending the invited schools using the SCQ-L. This method allowed for a more comprehensive assessment of the general population, including children who may not have been previously identified or suspected of having ASD. By adopting a population-based screening approach, we were able to capture a higher prevalence of ASD cases in our study. This strength should be considered in light of a low percentage of parents who completed SCQ-L and the low percentage of parents with children with positive SCQs who accepted the clinical assessment.

Our study's male to female prevalence ratio of 4.4:1 was consistent with the ratios reported in other studies conducted in the United States (4.5:1, [4]) and various European countries, such as South-Eastern France (4.0:1), Iceland (4.4:1), and Denmark (3.9:1, [12]). It is worth noting that the Italian ASDEU study conducted by Narzisi and colleagues [28] reported a higher male to female prevalence ratio of 5.2:1 compared to our overall ratio. However, when comparing the specific findings from the city of Pisa, located in the Center of Italy, the prevalence rate reported by Narzisi and colleagues was similar to our local prevalence rate in the Center area (5.3:1). These similarities suggest that there may be regional variations in ASD prevalence within Italy, and the differences in the male to female ratio could be influenced by geographical or environmental factors specific to each region. The integration of phase 2 (screening of the general population) with phase 1 (certified ASD diagnosis records of the MoE) permitted to identify 0.2% more cases with ASD that would not otherwise have been detected. Computing the incidence of new ASD diagnosis for the Italian population (N = 1,718,545) aged 7–9 years old on 1st January 2017 [19], the expected number of undiagnosed children with ASD was 2062.

Notably, the majority (89.5%, n = 17) of the children diagnosed with ASD and not registered with the MoE in our study exhibited preserved language skills, IQ within the average range, and had low support needs. This finding is consistent with the observation that children with milder or less apparent ASD symptoms often receive a diagnosis later in life [36]. Among the 19 children diagnosed with ASD, two of them also presented intellectual disability. In these specific cases, the children had previously received a diagnosis of intellectual disability but had not undergone a specific assessment for ASD. It is crucial to conduct comprehensive evaluations that include the assessment of ASD in individuals with coexisting intellectual disability to ensure accurate diagnoses and provide tailored intervention strategies. To optimize outcomes for children with ASD, it is essential to equip parents and teachers with targeted intervention tools [25]. The cases of ASD identified in this project have been integrated into the support and intervention network for ASD within the public Italian National Health System, ensuring that these children receive appropriate services and support for their ASD needs.

The overall school participation rate in the project was considered satisfactory, with 62.6% of schools participating. However, it was observed that schools in the Central area, specifically in Rome and its province, had lower participation rates (48.3%). This could be attributed to the proximity of these schools to major universities and research centers, as they may have already been involved in other research projects. On the other hand, schools in the South area exhibited the highest participation rate, reaching 80.6%. This could be due to the limited opportunities for schools in the South to participate in a national research screening program, likely because of their geographical distance from the inland and research institutions.

Overall, 45.5% (n = 16,293) of parents of children attending the invited schools participated by filling out the SCQ-L. The participation rate of parents was lower in the Centre area (36.7%) and South area (40.2%) compared to the North area (61.4%). This difference in participation could be attributed to the variation in availability of child and adolescent mental health services across different regions of Italy, with the North having higher availability compared to the Centre and South regions [6]. Parents in the North area may be more informed about the importance of early detection of NDD and more willing to participate in research projects related to this topic. It is concerning that the majority of the parents (60.6%) refused to have their children assessed for ASD despite their SCQ-L scores being above the cut-off. There could be several reasons for this refusal. Some parents may feel that their child does not require clinical evaluation because they do not perceive any social or learning difficulties. Despite explaining the benefits of obtaining a diagnosis at excellent ASD centers without incurring any costs, some parents may not grasp the significance of participating in this ASD prevalence study for the community and their own child. Stigma associated with an ASD diagnosis may also be a concern for some parents. Additionally, parents from rural areas may experience difficulties in accessing clinical centers located in urban areas for the assessment. Future studies should consider providing the option of conducting assessments at home or remotely when feasible. Efforts should also be made to improve the participation rates of schools and parents. This can be achieved through media campaigns that promote the project and emphasize its importance for the general population, including those not directly affected by ASD. Providing incentives such as awards for teachers who actively participate or promote the project and offering benefits to ASD organizations that decide to participate in research projects could also help enhance participation rates.

While this study employed a rigorous methodological protocol to determine the prevalence of ASD in Italy, it is important to acknowledge certain limitations. Firstly, it is possible that the prevalence rate obtained in this study is underestimated due to a high rate of parents (44.9%) who did not provide consent for the evaluation of their children when their SCQ-L scores exceeded the cut-off. Low parental participation rates in projects of this nature are common [13].

Secondly, there is a possibility that our screening process may have missed identifying some children at risk for ASD due to potential misunderstandings of the SCQ-L questions by parents [42]. To minimize the risk of false negatives, we conducted assessments on 20% of the sample. This additional assessment process aimed to identify any potential cases that might have been overlooked during the initial screening phase. Finally, this study encompassed both urban and rural areas near a national clinical referral center specializing in the ASD diagnosis in children, mitigating potential biases associated with service accessibility. Nonetheless, it is essential to acknowledge that various factors could contribute to the diversity in estimated prevalence rates, including social factors (e.g., family socio-professional category, family composition) and environmental factors (e.g., exposure to pollutants), which remained unaccounted for in the current study.

## Conclusion

The present study found an ASD prevalence of approximately one in 77 children among 7- to 9-year-olds attending schools in three regions of Italy. The study protocol utilized a combination of registry data and screening of the general population, coordinated by the Italian National Institute of Health. The findings of this study are valuable for informing policies aimed at monitoring service activities, enhancing their quality, and customizing them to better meet the needs of children with ASD and their families. As suggested by Lord and colleagues [22], establishing formal documentation and support systems within healthcare, education, and social care sectors is crucial for recognizing and addressing the unique challenges and inequalities faced by individuals with autism. The epidemiological data obtained from this study provide important insights for guiding such initiatives and improving the overall well-being of individuals with ASD.

## Data Availability

Sensitive data have been anonymized and protected following the EU General Data Protection Regulation [EU 2016/679]. This implies that data cannot be shared outside the ASDEU group.
